# Incidence of spontaneous subdural hematoma in incident cases of pulmonary arterial hypertension: a registry of cases occurring over a five-year period

**DOI:** 10.1590/S1806-37132015000100014

**Published:** 2015

**Authors:** Luis Felipe Lopes Prada, Francisca Gavilanes, Rogério Souza

**Affiliations:** University of São Paulo, School of Medicine, Hospital das Clínicas, São Paulo, Brazil, Heart Institute, University of São Paulo School of Medicine Hospital das Clínicas, São Paulo, Brazil; University of São Paulo, School of Medicine, Hospital das Clínicas, São Paulo, Brazil, Heart Institute, University of São Paulo School of Medicine Hospital das Clínicas, São Paulo, Brazil; University of São Paulo, School of Medicine, São Paulo, Brazil, University of São Paulo School of Medicine, São Paulo, Brazil

To the Editor:

Imatinib, a tyrosine-kinase inhibitor, has recently been tested to determine its safety and efficacy for the treatment of pulmonary arterial hypertension (PAH), specifically in a study entitled Imatinib in Pulmonary Arterial Hypertension, a Randomized, Efficacy Study (IMPRES).^(^ 1 ^)^ Experimental data suggest that imatinib plays a role in controlling pulmonary vascular remodeling, and this hypothesis had been previously tested in isolated case reports. ^(^ 2 ^)^ Nevertheless, the results of the IMPRES, a randomized, double-blind, placebo-controlled trial of imatinib mesylate as add-on therapy for pulmonary arterial hypertension, clearly demonstrated an increase in the occurrence of one severe side effect-spontaneous subdural hematoma.^(^ 1 ^)^ The authors reported eight separate cases in which patients developed spontaneous subdural hematoma: two during the core study (in which 103 patients were enrolled in the treatment group) and six during the open-label, long-term extension study (in which 144 patients opted to be treated with imatinib). All of the patients were using oral anticoagulants at target levels.

In patients with chronic myeloid leukemia, the first study to investigate the efficacy of imatinib showed no spontaneous subdural hematoma but did identify thrombocytopenia in 4-24% of the patients, depending on the dosage.^(^ 3 ^)^ After the use of imatinib became widespread, there were some reports of spontaneous bleeding and (more rarely) spontaneous subdural hematoma.^(^ 4 ^)^

A recent review of two randomized controlled trials of targeted therapies in PAH, collectively involving 564 patients, reported the occurrence of two events of spontaneous subdural hematoma among those patients, which translates to an incidence of 0.3% (95% CI: 0.1-1.3).^(^ 5 ^)^ In both of those cases, the patients were using oral anticoagulants. The risk of bleeding in PAH patients was further evaluated in a study involving 218 patients with chronic thromboembolic pulmonary hypertension, connective tissue disease-associated PAH, and idiopathic PAH.^(^ 6 ^)^ All of the patients evaluated in that study were receiving vitamin K antagonists. The authors found that the incidence of bleeding was highest in the patients with connective tissue disease-associated PAH, although central nervous system bleeding occurred in only one case (0.4%).

We have recently created a registry of incident cases of PAH treated at a large referral center in Brazil over a five-year period (2008-2013).^(^ 7 ^,^ 8 ^)^ During that period, 178 newly diagnosed cases were included in the registry. During follow-up, two patients presented with spontaneous subdural hematoma, corresponding to an incidence of 1.1% (95% CI: 0.3-4.0): one was a female patient with idiopathic PAH (baseline mean pulmonary artery pressure of 50 mmHg; cardiac output of 4.3 L/min) who was using bosentan, and one was a male patient with schistosomiasis-associated PAH (baseline mean pulmonary artery pressure of 55 mmHg; cardiac output of 2.71 L/min) who was using sildenafil. Neither of those patients were using an oral anticoagulant. 

Our data provide the first prospectively collected data on the incidence of spontaneous subdural hematoma in patients with PAH managed at a tertiary referral center. Our results underscore the assertion that the events reported in the IMPRES are not trivial and truly represent a major cause for concern regarding the safety of imatinib for use in PAH. 
